# Multimerization results in formation of re-bindable metabolites: A proof of concept study with FSC-based minigastrin imaging probes targeting CCK2R expression

**DOI:** 10.1371/journal.pone.0201224

**Published:** 2018-07-30

**Authors:** Dominik Summer, Andrea Kroess, Rudolf Woerndle, Christine Rangger, Maximilian Klingler, Hubertus Haas, Leopold Kremser, Herbert H. Lindner, Elisabeth von Guggenberg, Clemens Decristoforo

**Affiliations:** 1 Department of Nuclear Medicine, Medical University of Innsbruck, Innsbruck, Austria; 2 Division of Molecular Biology, Biocenter, Medical University of Innsbruck, Innsbruck, Austria; 3 Division of Clinical Biochemistry, Biocenter, Medical University of Innsbruck, Innsbruck Austria; University of California Berkeley, UNITED STATES

## Abstract

Positron emission tomography (PET) with radiolabelled peptide-based tracers has attracted great interest in oncology over the past decades. The success of imaging is closely related to sufficient uptake of the radiotracer in malignant tissue and for this sufficient biological half-life, particularly in the bloodstream, is mandatory. Fast enzymatic degradation during circulation leading to insufficient imaging abilities of peptide-based radioligands remains a major issue. The design of multimeric constructs, bearing multiple targeting moieties, has been widely applied to improve target interaction. This concept may also be applied to prolong the biological half-life of peptide-based radiopharmaceuticals as enzymatic degradation can result in formation of metabolites still capable to interact with the target binding site. In this study we aimed to identify such metabolites and therefore we utilized the siderophore-based bifunctional chelator fusarinine C (FSC) for the design of novel mono- and multimeric constructs, bearing minigastrin (MG) analogues as targeting moieties to address cholecystokinin-2 receptors (CCK2R) which are overexpressed in a variety of cancerous diseases and are well known for fast enzymatic degradation, particularly for truncated des-(Glu)_5_-MG members, such as MG11. FSC-based imaging probes were radiolabelled with gallium-68 and characterized *in vitro* (logD, protein binding, affinity and cell-uptake studies, stability and metabolite studies, as well as generation of corresponding metabolites by artificial enzymatic degradation) and *in vivo* (biodistribution in A431-CCK2R/A431-mock tumour xenografted BALB/c nude mice and stability in blood of living BALB/c mice and analysis of corresponding organ homogenates and urine to identify degradation products). In summary, multimerization was accompanied by partial improvement towards targeting abilities. Identified metabolites formed by artificial enzymatic cleavage of trimeric FSC-MG conjugates *in vitro* contained intact binding sequences for the receptor. Furthermore, the ^68^Ga-labelled trimers exhibiting increasing uptake of radioligand in tumour tissue over time and improved *in vivo* stability in blood samples of living animals of the trimers compared to corresponding mono- and dimers, strongly supporting our hypothesis.

## Introduction

**Fig 1:**
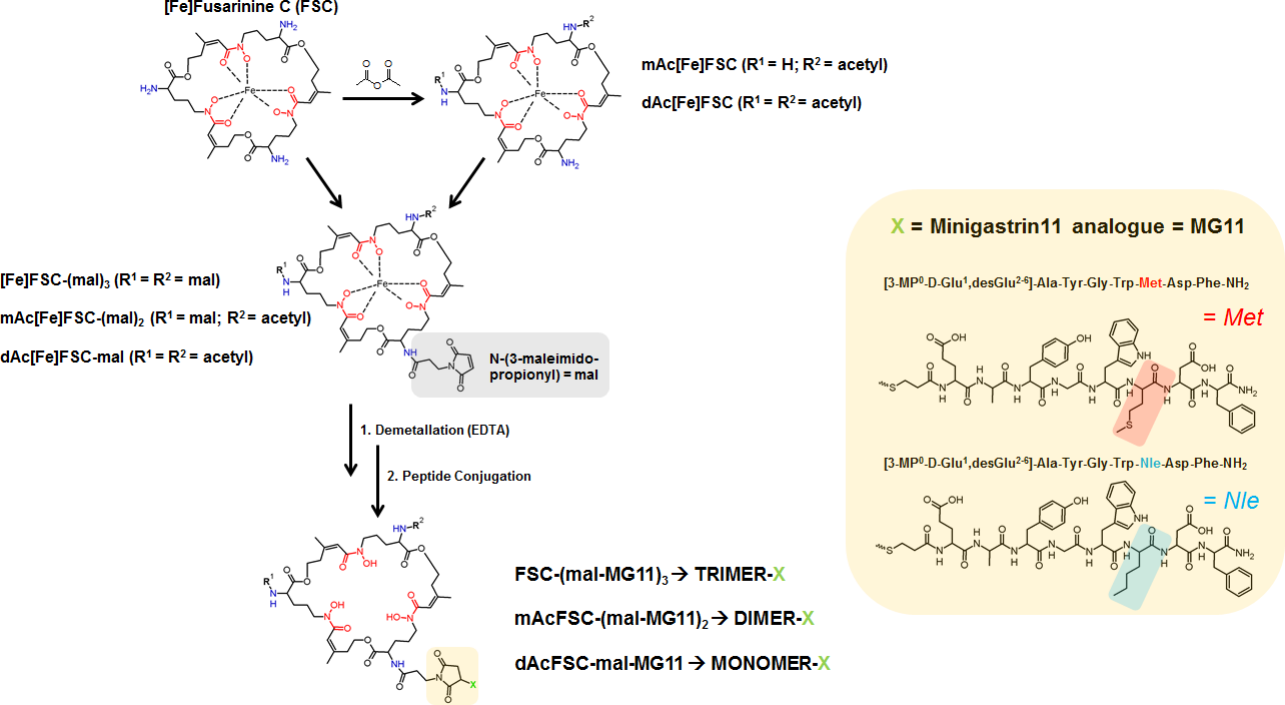
Synthesis of FSC-based mono- and multivalent conjugates. Schematic overview on the synthetical pathway of mono- and multimeric FSC-based minigastrin bioconjugates (stereochemistry omitted).

Positron emission tomography (PET) imaging with peptide receptor targeting probes is used in oncology for many decades and has anchored itself as convenient non-invasive method to identify malignancies with high resolution in nuclear medicine. Therefore, radiopharmaceutical engineering of novel peptide-based tracers with potentially improved targeting properties plays a fundamental role for *in vivo* imaging of cancerous diseases[1]. Radiotracers being intravenously injected face quite inhospitable environment among their journey through the bloodstream before being distributed to the target interaction site. Especially degradation by soluble or membrane bound enzymes mainly in blood liver and kidney [2], can cause severe damage to the peptide-based tracer and lead to decreased biological stability resulting in significant loss of receptor affinity and insufficient imaging abilities. Several strategies e.g. amino acid exchange (L-amino acids by their corresponding D-amino acid) peptide cyclization and co-injection of neutral endopeptidase inhibitor, have been implemented to prevent enzymatic cleavage and prolong the biological half-life of peptide-based pharmaceuticals[2][3][4][5][6]. Another approach would be the design of multimeric constructs improving avidity and affinity, for review see Carlucci and co-workers[7]. Several studies reported on beneficial effects by utilizing the concept of multivalency for targeted PET imaging in oncology[8][9][10][11][12][13]. The effects of an improved metabolic stability by applying this concept have scarcely been reported. In particular, it has never been investigated if the beneficial effect of multimerization could also be a result of retained binding of a multimeric molecule even after initial metabolization. We recently reported on ^68^Ga- and ^89^Zr-labelled mono-, di- and trimeric minigastrin (MG) analogues utilizing fusarinine C (FSC) as bifunctional chelator (BFC) for targeting cholecystokinin-2 receptor (CCK2R) expression which is involved in various malignancies as medullary thyroid carcinoma (MTC), small cell lung cancer (SCLC) and stromal ovarian cancer[14]. The multivalent FSC-based imaging probes showed partly improved targeting properties *in vitro* and *in vivo* 1 h p.i. However, in this study we observed at later time points, 24 h p.i. respectively, that the beneficial effect of the trimeric conjugate was more pronounced in comparison to the mono- and divalent imaging probe and we therefore assumed that this may be related to the formation of metabolites still retaining their receptor binding properties[15]. In order to confirm this hypothesis we report here on the synthesis of a second series of mono- and multimeric FSC-based MG analogues (both series are shown in Fig 1) and their evaluation *in vitro* and *in vivo*. *In vitro* studies included determination of logD, protein binding, IC_50_, cell-uptake and stability in metabolically active tissue. *In vivo* experiments included biodistribution in double tumour xenografted BALB/C nude mice and blood stability in non-tumour xenografted BALB/C mice. In particular we focussed on the identification of metabolites formed by enzymatic degradation both *in vitro* and *in vivo* to identify potential compounds with retained receptor targeting properties.

## Experimental section

### Ethics statement

All animal experiments were performed with approval of the National Committee for Animal Care of the Austrian Federal Ministry of Science, Research and Economy. (BMWF-66.011/000604-II/3b/2012 and BMWFW-66.011/ 0049-WF/II/3b/2014) and were in compliance with the Austrian animal protection laws. Housing conditions at the Central Laboratory Animal Facility of Medical University Innsbruck were maintained at constant temperature and relative humidity with 12/12 h light/dark cycle, animals were fed ad libitum and fresh water was constantly available, animal welfare was monitored at least daily.

### Instrumentation

#### Analytical [radio]-RP-HPLC

Reversed-phase high-performance liquid chromatography analysis was performed on an UltiMate 3000 RS UHPLC pump, UltiMate 3000 autosampler, Ultimate 3000 column compartment (25°C oven temperature), UltiMate 3000 Variable Wavelength Detector (Dionex, Germering, Germany; UV detection at λ = 220 nm) and a radio detector (GabiStar, Raytest; Straubenhardt, Germany). A Jupiter 5μm C_18_ 300Å 150 × 4.6 mm (Phenomenex Ltd. Aschaffenburg, Germany) column was used with Acetonitrile (ACN)/H_2_O/ 0.1% trifluoroacetic acid (TFA) as mobile phase: **gradient (synthesis):** flow rate of 1 mL/min; gradient: 0.0–3.0 min 0% ACN, 3.0–5.0 min 0–30% ACN, 5.0–20.0 min 30–60% ACN, 20.0–25.0 min 60–80% ACN, 26.0–30.0 min 80–0% ACN. **Gradient (radiolabelling, stability & metabolite studies):** flow rate of 1.4 mL/min; gradient: 0.0–1.5 min 0% ACN, 1.5–11.5 min 30–50% ACN, 11.5–13.5 min 0% ACN.

#### Preparative RP-HPLC

Sample purification was carried out on a RP-HPLC [Gilson 322 Pump with a Gilson UV/VIS-155 detector (UV detection at λ = 220 nm) using a PrepFC™ automatic fraction collector (Gilson, Middleton, WI, USA)] using Eurosil Bioselect Vertex Plus 30 x 8 mm 5 μm C_18_A 300Å pre-column and Eurosil Bioselect Vertex Plus 300 x 8 mm 5 μm C_18_A 300Å column (Knauer, Berlin, Germany) with a flow rate of 2 mL/min and a mobile phase with following ACN/H_2_O/ 0.1% TFA gradient: 0.0–1.0 min 20% ACN, 1.0–26.0 min 20–80% ACN, 26.0–28.0 min 80% ACN, 28.0–30.0 min 20% ACN.

#### Mass analysis (MALDI-TOF-MS)

Mass spectrometry was conducted on a Bruker microflex bench-top MALDI-TOF MS (Bruker Daltonics, Bremen, Germany). Samples were prepared via dried-droplet on a micro scout target (MSP96 target ground steel BC, Bruker Daltonics) using α-cyano-4-hydroxy-cinnamic acid (HCCA, Sigma-Aldrich, Handels GmbH, Vienna, Austria) as matrix. Recorded data, summation of 800 shots per spot, was processed using Flex Analysis 2.4 software.

### Precursor preparation

#### Synthesis of thiol functionalized MG11 analogues (MG-X-SH)

The synthesis was carried out as previously described[16] following straight forward solid-phase peptide synthesis. Briefly, rink amide MBHA resin (100–200 mesh, 62.5 μmol), N-terminal 9-Fluorenylmethoxycarbonyl (Fmoc) and side-chain protected amino acids (Boc-Trp, OtBu for Asp, Tyr and D-Glu) from Novabiochem (La Jolla, CA, USA) and pre-activation strategy with O-(7-azabenzotriazol-1-yl)-1,1,3,3-tetramethyluronium-hexafluorophosphate (HATU) and 1-Hydroxy-7-azabenzotriazole (HOAt) both from GenScript Corporation (Piscataway, NJ, USA) was applied. Amino acid conjugation was performed with a threefold molar excess (187.5 μmol) and Kaiser test was used to monitor coupling efficiency. The final step before cleavage from the resin was the conjugation of (3-MP^0^) 3-(tritylthio)propanoic acid (Bachem, Bubendorf, Switzerland) to introduce a thiol functionality. Finally, cleavage from the resin and side chain deprotection was carried out under acidic conditions (TFA/Triisopropylsilane/H_2_O (v/v/v; 95/2.5/2.5)) followed by precipitation of the crude product in ice-cold diethyl ether. The crude peptides were dissolved in methanol and were purified by preparative RP-HPLC (MG-Met-SH t_R_ = 21.6 min, MG-Nle-SH t_R_ = 23.1 min) to give a colourless powder after lyophilization.

**MG-Met-SH:** [= 3-MP^0^-[D-Glu^1^,desGlu^2-6^]-Ala-Tyr-Gly-Trp-Met-Asp-Phe-NH_2_] 37.6 mg [34.0 μmol, 54%]; analytical data: RP-HPLC t_R_ = 12.8 min; m/z [M+Na]+ = 1128.23; [M+K]+ = 1144.36 [C_51_H_64_N_10_O_14_S_2_; exact mass: 1105.24 (calculated)].

**MG-Nle-SH:** [= 3-MP^0^-[D-Glu^1^,desGlu^2-6^]-Ala-Tyr-Gly-Trp-Nle-Asp-Phe-NH_2_] 32.1 mg [29.5 μmol, 47%]; analytical data: RP-HPLC t_R_ = 13.6 min; m/z [M+Na]+ = 1110.02; [M+K]+ = 1126.02 [C_52_H_66_N_10_O_14_S; exact mass: 1087.20 (calculated)].

#### Extraction and derivatization of [Fe]Fusarinine C

[Fe]Fusarinine C ([Fe]FSC) was obtained from fungal culture, was modified by acetylation and functionalized with maleimide (mal) for site specific conjugation of the targeting moieties as previously described[15].

[Fe]FSC: analytical data: RP-HPLC t_R_ = 8.1 min; m/z [M+H]+ = 780.86 [C_33_H_51_FeN_6_O_12_; exact mass: 779.63 (calculated)] → FSC-mal_3_: analytical data: RP-HPLC t_R_ = 12.6 min; m/z [M+Na]+ = 1203.27 [C_54_H_69_N_9_O_21_; exact mass: 1180.17 (calculated)].

mAc[Fe]FSC: analytical data: RP-HPLC t_R_ = 8.4 min; m/z [M+H]+ = 822.90 [C_35_H_53_FeN_6_O_13_; exact mass: 821.67 (calculated)] → mAcFSC-mal_2_: analytical data: RP-HPLC t_R_ = 11.4 min; m/z [M+H]+ = 1072.21 [C_49_H_66_N_8_O_19_; exact mass: 1071.09 (calculated)].

dAc[Fe]FSC: analytical data: RP-HPLC t_R_ = 8.8 min; m/z [M+H]+ = 864.95 [C_37_H_55_FeN_6_O_14_; exact mass: 863.71 (calculated)] → dAcFSC-mal: analytical data: RP-HPLC t_R_ = 10.8 min; m/z [M+H]+ = 963.03 [C_44_H_63_N_7_O_17_; exact mass: 962.01 (calculated)].

#### Conjugation of targeting vector(s)

To conjugate thiol functionalized MG analogues site specifically 1 mg of each, FSC-mal_3_ (0.85 μmol), mAcFSC-mal_2_ (0.93 μmol) and dAcFSC-mal (1.04 μmol) were dissolved in phosphate buffered saline (PBS, pH 7.2) and either MG-Met-SH or MG-Nle-SH were added in 1.5 to 4 fold molar excess. After 2 h of continuous stirring at room temperature the bioconjugates were isolated in excellent purity (>95%) by preparative RP-HPLC [Monomer-Met/Nle t_R_ = 18.7/19.6 min; Dimer-Met/Nle t_R_ = 20.6/22.1 min; Trimer-Met/Nle t_R_ = 22.9/25.0 min] giving colourless solids after lyophilisation.

Monomer-Met 1.8 mg [0.87 μmol, 84%]; analytical data: RP-HPLC t_R_ = 13.6 min; m/z [M+Na]+ = 2090.99 [C_95_H_127_N_17_O_31_S_2_; exact mass: 2067.25 (calculated)].

Monomer-Nle 1.5 mg [0.73 μmol, 70%]; analytical data: RP-HPLC t_R_ = 14.3 min; m/z [M+H]+ = 2050.25 [C_96_H_129_N_17_O_31_S; exact mass: 2049.21 (calculated)].

Dimer-Met 2.2 mg [0.67 μmol, 72%]; analytical data: RP-HPLC t_R_ = 15.7 min; m/z [M+Na]+ = 3304.66 [C_151_H_194_N_28_O_47_S_4_; exact mass: 3281.58 (calculated)].

Dimer-Nle 2.4 mg [0.74 μmol, 79%]; analytical data: RP-HPLC t_R_ = 16.5 min; m/z [M+H]+ = 3246.61 [C_153_H_198_N_28_O_47_S_2_; exact mass: 3245.50 (calculated)].

Trimer-Met 2.4 mg [0.53 μmol, 63%]; analytical data: RP-HPLC t_R_ = 16.9 min; m/z [M+H]+ = 4496.65 [C_207_H_261_N_39_O_63_S_6_; exact mass: 4495.90 (calculated)].

Trimer-Nle 1.9 mg [0.43 μmol, 50%]; analytical data: RP-HPLC t_R_ = 17.8 min; m/z [M+H]+ = 4441.83 [C_210_H_267_N_39_O_63_S_3_; exact mass: 4441.79 (calculated)].

#### Iron chelated trimers

To obtain iron containing trimers for metabolite studies 1 mg of the corresponding metal free trimer was dissolved in PBS and after addition of 20 μL FeCl_3_ solution (100 mM) the reaction mixture was purified by preparative RP-HPLC ([Fe]Trimer-Met/Nle t_R_ = 23.1/25.2 min) to give red brown coloured solids after freeze drying.

[Fe]Trimer-Met 0.93 mg [0.20 μmol, 92%]; analytical data: RP-HPLC t_R_ = 17.2 min; m/z [M+H]+ = 4549.93 [C_207_H_258_FeN_39_O_63_S_6_; exact mass: 4548.72 (calculated)].

[Fe]Trimer-Nle 0.81 mg [0.18 μmol, 80%]; analytical data: RP-HPLC t_R_ = 18.1 min; m/z [M+H]+ = 4495.27 [C_210_H_264_FeN_39_O_63_S_3_; exact mass: 4494.61 (calculated)].

### Radiolabelling with gallium-68

Gallium-68 chloride (^68^GaCl_3_) solution was obtained by fractioned elution (~300 MBq/1 mL) of a commercial available ^68^Ga/^68^Ge-generator (IGG100, Eckert & Ziegler Isotope products, Berlin, Germany) with 0.1 M hydrochloric acid (HCl, Rotem Industries, Israel). For Radiolabelling 100 to 500 μL eluate were mixed with sodium acetate solution (1.14 M, 20 μL/100 μL eulate) to adjust a pH of 4.5, followed by subsequent addition of the conjugate (5–10 nmol). After incubation for 5 to 15 min at ambient temperature the reaction was analysed by *radio*-RP-HPLC as well as *radio*-ITLC (TLC-SG strips: Varian, Lake Forest, CA, USA; solvent: 0.1 M sodium citrate solution (pH 5); TLC scanner: Scan-RAM™, LabLogic Systems Ltd., Sheffield, UK).

### Cell culture

For cell experiments two variants of the human epidermoid carcinoma cell line (A431) were used. A431-CCK2R are stably transfected with the full coding sequence for the human CCK2R and the other with the negative control cell line is transfected with the empty vector alone (A431-mock)[17]. Both cell lines were a kind gift from Dr. L. Aloj and were cultured in Dulbecco’s Modified Eagle’s Medium (DMEM) with 10% v/v fetal bovine serum (FBS) and 1% v/v penicillin-streptomycin-glutamin (PSG) solution media (Gibco, Invitrogen Corporation, Paisley, UK) as additives.

### *In vitro* characterization

#### Distribution coefficient (logD)

To determine the distribution of the ^68^Ga-radiolabelled conjugates between an organic and aqueous layer aliquots (50 μL) of the tracers (10 μM) were diluted in 450 μL PBS and after adding 500 μL octanol the mixture was vortexed at 1400 rpm (MS 3 basic vortexer, IKA, Staufen, Germany) for 15 min at RT followed by centrifugation for 2 min at 4500 rpm. Subsequently, aliquots (100 μL) of both layers were collected and measured in the gamma counter (Wizard2 3”, Perkin Elmer, Waltham, MA, USA) followed by logD calculation using Excel (n = 3, six replicates).

#### Protein binding

The ability to bind to serum proteins was conducted via size exclusion chromatography using MicroSpin G-50 columns (Sephadex G-50, GE Healthcare, Vienna, Austria) according to manufacturer’s protocol. Therefore aliquots (50 μL) of the radioligand solution (10 μM) were incubated in 450 μL PBS as control and 450 μL freshly prepared human serum and were maintained at 37°C. After 1, 2, and 4 h aliquots (25 μL) were transferred to the column and after centrifugation (2 min, 2000 rcf) the column containing the free conjugate and the eluate containing the protein-bound conjugate were measured in the gamma counter followed by calculating the percentage between both fractions.

#### Receptor binding affinity studies (IC_50_)

Binding affinity studies on whole A431-CCK2R cells were performed as previously described[16]. Briefly, 100 μL of cell suspension (5 × 10^6^ cells/mL) was added to each well of a 96-well MultiScreen Filter Plate HTS (1 μm glass fiber filter, Merck Millipore, Darmstadt, Germany). Hereafter competitor solution was added with increasing concentrations [0.001−1.000 nM] and after 10 min at RT human [Leu^15^]-gastrin I radiolabelled with iodine-125 as described in [16] was added (4.5 × 10^4^ cpm) and the plate was maintained under shaking conditions (Compact shaker KS-15 control, 200/min) at ambient temperature. After 1 h each well was washed twice with 200 μL TRIS-buffered saline (pH 7.3), filters were collected and measured in the gamma counter followed by nonlinear curve fitting with Origin 6.1 software (Northampton, MA, USA) to calculate the IC_50_ values.

#### CCK-2 receptor internalization assay

Cellular processing of the ^68^Ga-labelled radioligands on A431-CCK2R cells was conducted following previously published protocol[15]. Briefly, 12-well plates (Nunc, Thermo Scientific) were seeded with A431- CCK2R cells (2×10^6^ cells per well) and left to grow over night. After washing and volume adjustment to 1.2 mL with DMEM, half of the plate was blocked by adding 100-fold molar excess of pentagastrin prior to addition of the radioligand (1 nM). After 1 and 2 h incubation at 37°C the cells were washed with ice-cold medium (= wash fraction), with ice-cold 0.05 M glycine buffer (pH 2.8) (= membrane bound fraction) and finally detached with 2 M sodium hydroxide (= internalized fraction). Collected fractions were measured in the gamma counter to determine the percentage of cell associated radioactivity in relation to the total activity added. In case of trimeric conjugates additional experiments were performed to specify the unspecifically bound amount of radioligand by repeating this assay but using A431-mock (receptor negative) cells instead.

#### Stability studies in organ homogenates

To determine the stability in metabolic active tissue liver and kidneys were excised from CO_2_ asphyxiated female rats (CD^®^ (Sprague Dawley) IGS rats, Charles River Laboratories, Sulzfeld, Germany) and the organs were immediately frozen at -80°C. On the day of the experiment organs were thawed and homogenised in 20 mM HEPES buffer (pH 7.3) with an UltraTurrax T8 homogenator for 2 min at ambient temperature. Subsequently, the homogenates were diluted with HEPES buffer to a final concentration of 1% and 10% (w/v) and were maintained under an argon stream for 1 min. Conjugates radiolabeled with gallium-68 were incubated with homogenates at 37°C and a constant peptide concentration of 4.4 μM. After 2, 4, 6, 8, 10, 15 min aliquots (100 μL) of the homogenate were mixed with 0.1% TFA/ACN (1:1) and the mixture was centrifuged at 14.000 rpm for 5 min followed by radio-RP-HPLC analysis of the supernatant.

#### Enzymatic degradation of the radiopeptides in human serum

To determine the formation of metabolites of the trimeric conjugates the radiolabelling solution (4.4 μM) was diluted with PBS to a final volume of 1 mL. An aliquot of 50 μL was mixed with 450 μL freshly prepared human serum and the samples were incubated at 37°C. After 1, 2 and 4 h an aliquot (100 μL) was mixed with 0.1% TFA/ACN (1:1), centrifuged for 2 min at 14.000 rpm followed by subsequent analysis of the supernatant by radio-RP-HPLC.

#### Artificial metabolite formation by enzymatic degradation

To generate artificial metabolites of the ^68^Ga-labelled trimeric conjugates the radiolabelling solution was neutralized with sodium carbonate solution (Na_2_CO_3_, 1 M), diluted with PBS to a final concentration of 4.4 μM and after adding 10 mU of peptidase (protease type 1 from bovine pancreas, Sigma Aldrich, Vienna, Austria) the mixture was maintained at 37°C. Samples (25 μL) were taken at different time points (5, 10, 60 and 120 min), 0.1% TFA/methanol (1:1) was added to quench the enzymatic reaction followed by subsequent radio-RP-HPLC analysis.

#### Preparation of metabolites for isolation

The trimeric peptides (100 μg) were labelled with gallium following the procedure described above but using 100 fold molar excess of gallium chloride (GaCl_3_ dissolved in HCl) instead of generator eluate. After neutralization of the solution with Na_2_CO_3_ solution (1 M), addition of 1 mU of peptidase and incubation at 37°C the degradation profile was monitored by RP-HPLC. For practical reasons i.e. the red brown colour of iron chelated trimeric conjugates and corresponding metabolites isolation of artificial metabolites for identification was performed by enzymatic degradation of the Fe-Trimers. Therefore the iron chelated trimeric peptides were dissolved in PBS (1 mg/mL), 1 mU of peptidase was added and the degradation profile was monitored by RP-HPLC. After 2 h incubation at 37°C 0.1% TFA/methanol was added to stop the enzymatic degradation and the mixture was frozen in liquid nitrogen and freeze dried overnight. The crude residue was dissolved in 200 μL H_2_O/methanol (2:1) and an aliquot (100 μL) was injected to the analytical RP-HPLC followed by manual collection of the metabolites. Collected metabolite fractions were reinjected to ensure purity, were lyophilized for sample concentration and analysed by mass spectrometry.

#### Computational model for metabolite verification

A simplified model for the determination of the molecular weight of metabolites consists of a single FSC-core with three potentially single directional reduced peptide sequences. The model has a maximal combinatorial number of 165 different molecular weights. This increases linear with the number of mutations occurring on the core. The possible molecular weights can be enumerated and filtered to a certain error range but the error range has to cover the expected measuring error—at minimum. To ensure an efficient calculation a constraint solver using fixed point notation was used. Therefore an algorithm was programmed accordingly and fed with the molecular weights of all amino acids and FSC-based scaffolds. Additionally, loss of metal and metal adduct formation during ionisation process was included in the calculation and an error range of ± 10 (Da) was allowed in this case to cover all possible solutions within that error range.

### *In vivo* characterization

#### *In vivo* stability

*In vivo* stability studies were performed with 5 weeks old female BALB/c mice (in-house breed, ZVTA Innsbruck, Dr. Hermann Dietrich). To determine the stability the labelling solution was neutralized to pH 7.2 by adding Na_2_CO_3_ (1M) followed by intravenous injection of the ^68^Ga-labelled conjugates (1.5 nmol, 15 MBq). Mice were euthanised 5 min post injection (p.i.) by cervical dislocation and blood samples were taken by heart puncture and collected in a heparinised Eppendorf tube. Furthermore, urine was collected and liver and kidneys were excised and homogenated as described above to a homogenate concentration of 15%. Aliquots (50 μL) of the collected samples were immediately precipitated by adding 0.1% TFA/ACN (1:1 v/v) and the supernatant was diluted with H_2_O (1:1) and analysed by radio-RP-HPLC.

#### *Ex vivo* biodistribution

Ex *vivo* biodistribution studies were conducted using female 8−10 weeks old athymic BALB/c nude mice (Charles River Laboratories, Sulzfeld, Germany). Tumour xenografts were induced by subcutaneous injection of 2 × 10^6^ A431-CCK2R cells (receptor positive) in the right and 2 × 10^6^ A431-mock cells (receptor negative) in the left flank and tumours were allowed to grow until they had reached a volume of 0.3–0.6 cm^3^.To evaluate the biodistribution profile double tumour xenografted BALB/c nude mice (n = 3) were intravenously injected with 1–2 MBq of ^68^Ga-labelled peptides (0.1–0.2 nmol). The animals were sacrificed by cervical dislocation after 1 h and 4 h in case of the trimer. The collected samples were measured in the gamma counter and the results were calculated as percentage of injected activity per gram tissue (% IA/g).

### Statistical analysis

Statistical analysis was performed using the Student’s t test with P value < 0.05 indicating significance.

## Results and discussion

### Precursor synthesis

Synthesis of MG-analogues, extraction, derivatization and demetallation of the chelating backbone was straight forward following a well-established in house protocol. Conjugation of up to three targeting vectors was conducted site specifically utilizing maleimide-thiol crosslink reaction. Resulting conjugates were obtained in good yield, excellent chemical purity (> 95%; analytical RP-HPLC, UV absorption at λ = 220 nm) and mass analysis was in good agreement with the calculated values.

### Radiolabelling

**Fig 2:**
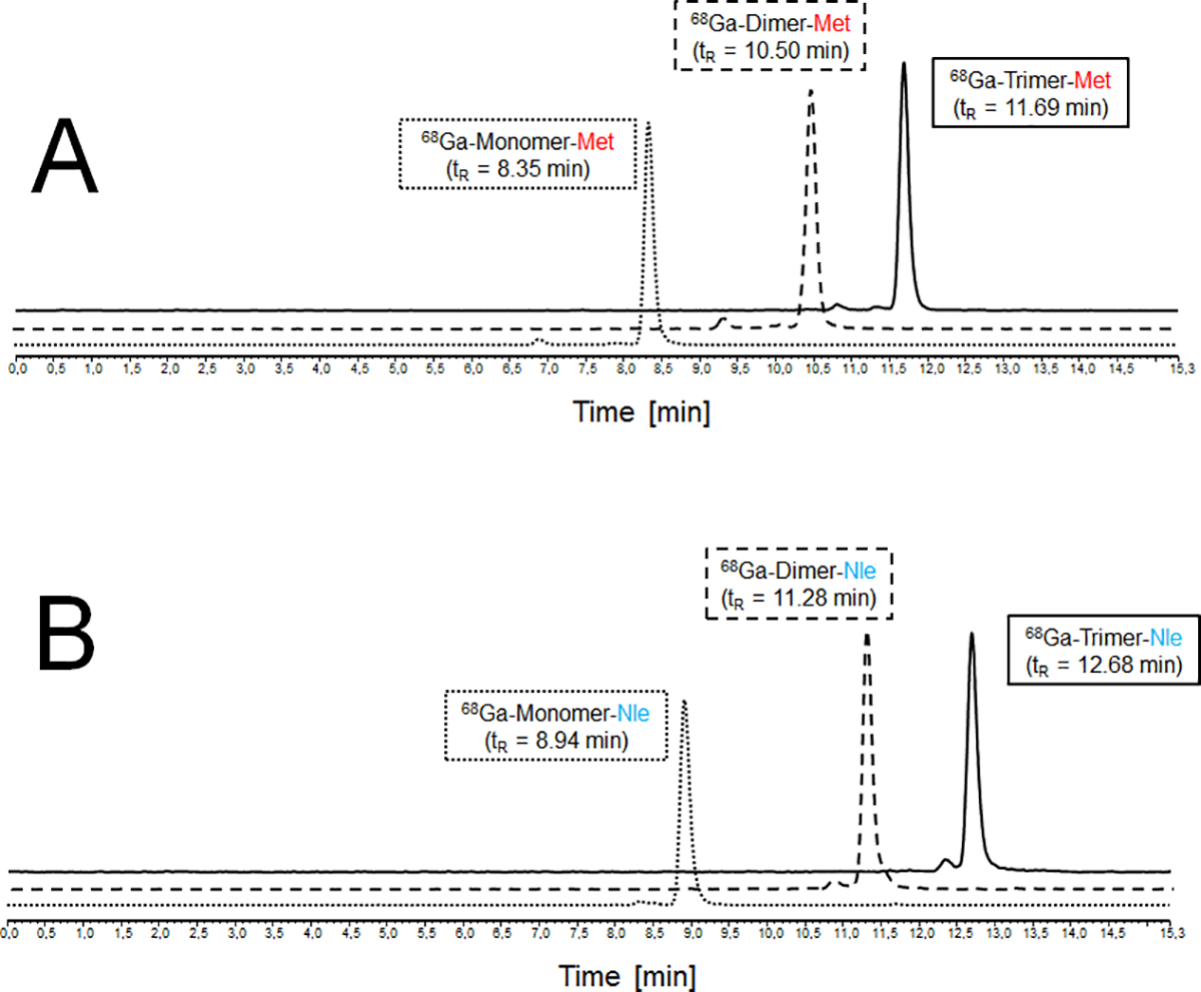
Radiolabelling of FSC-based mono- and multimers with gallium-68. Representative radio-RP-HPLC chromatograms of ^68^Ga-labelled mono- and multimers with Met-sequence (A) and Nle-sequence (B).

Mono- and multimeric FSC-based MG conjugates were quantitatively labelled with gallium-68 at ambient temperature within minutes and resulting radiotracers showed sufficient radiochemical purity (> 94%) to be used for further experiment without purification (Fig 2).

### *In vitro* evaluation

The distribution coefficient expressed as logD and the ability of ^68^Ga-labeled mono- and multimeric peptides to bind to proteins in human serum is summarized in Table 1. Increasing the grade of multimerization is accompanied by lower hydrophilicity and increased protein binding. This trend is quite consistent with previous results[15] and Nle-derivatives in general showed somewhat higher lipophilicity and protein binding compared to the ^68^Ga-labelled Met-derivatives.

**Table 1:** Distribution coefficient and protein binding of mono- and multimeric conjugates of two different MG analogues (-Met/Nle) radiolabelled with gallium-68.

		^68^Ga-labelled					
		Monomer-Met[15]	Dimer-Met[15]	Trimer-Met[15]	Monomer-Nle	Dimer-Nle	Trimer-Nle
Distribution coefficient	logD (pH 7.4)	-2.99 ± 0.02	-2.38 ± 0.04	-2.20 ± 0.07	-2.69 ± 0.14	-2.05 ± 0.19	-1.80 ± 0.16
Protein binding (%)	1 h	3.71 ± 0.86	15.94 ± 1.25	40.69 ± 0.93	9.95 ± 1.29	44.75 ± 1.05	54.46 ± 2.33
	2 h	4.16 ± 0.22	18.77 ± 2.93	44.33 ± 1.41	10.95 ± 0.91	43.57 ± 0.35	53.53 ± 2.75
	4 h	3.16 ± 0.49	17.86 ± 0.32	48.32 ± 1.71	13.40 ± 2.44	49.45 ± 3.17	51.16 ± 2.05

Data are presented as mean ± SD (n = 3)

Receptor affinity studies (Table 2) showed values in the low nanomolar range for all mono- and multimeric targeting probes indicating high binding affinity to the CCK2 receptor evaluated on whole A431-CCK2R cells. Nle-derivatized targeting probes showed generally a lower affinity compared to the Met-counterparts but both series of mono- and multimers show the same trend as the receptor affinity of the dimer improved while the trimer remained comparable to the monomeric conjugate. Furthermore, the improvement of the divalent probe is substantiated by a previous report where a DOTA-based divalent probe (MGD5) also showed improved affinity compared to the corresponding monomer (APH070) regardless of varying experimental setup[18].

**Table 2:** Receptor affinity studies (IC_50_) with ^nat^Ga-bound peptides as competitor and human ^125^I-[Leu^15^]-Gastrin as radioligand on whole A431-CCK2R cells.

	^nat^Ga-labelled					
	Monomer-Met[15]	Dimer-Met[15]	Trimer-Met[15]	Monomer-Nle	Dimer-Nle	Trimer-Nle
IC_50_ (nM)	9.71 ± 3.55	0.85 ± 0.15	8.33 ± 2.20	12.3 ± 4.31	2.30 ± 0.87	20.9 ± 1.13

Data are presented as mean ± SD (n = 6)

**Fig 3:**
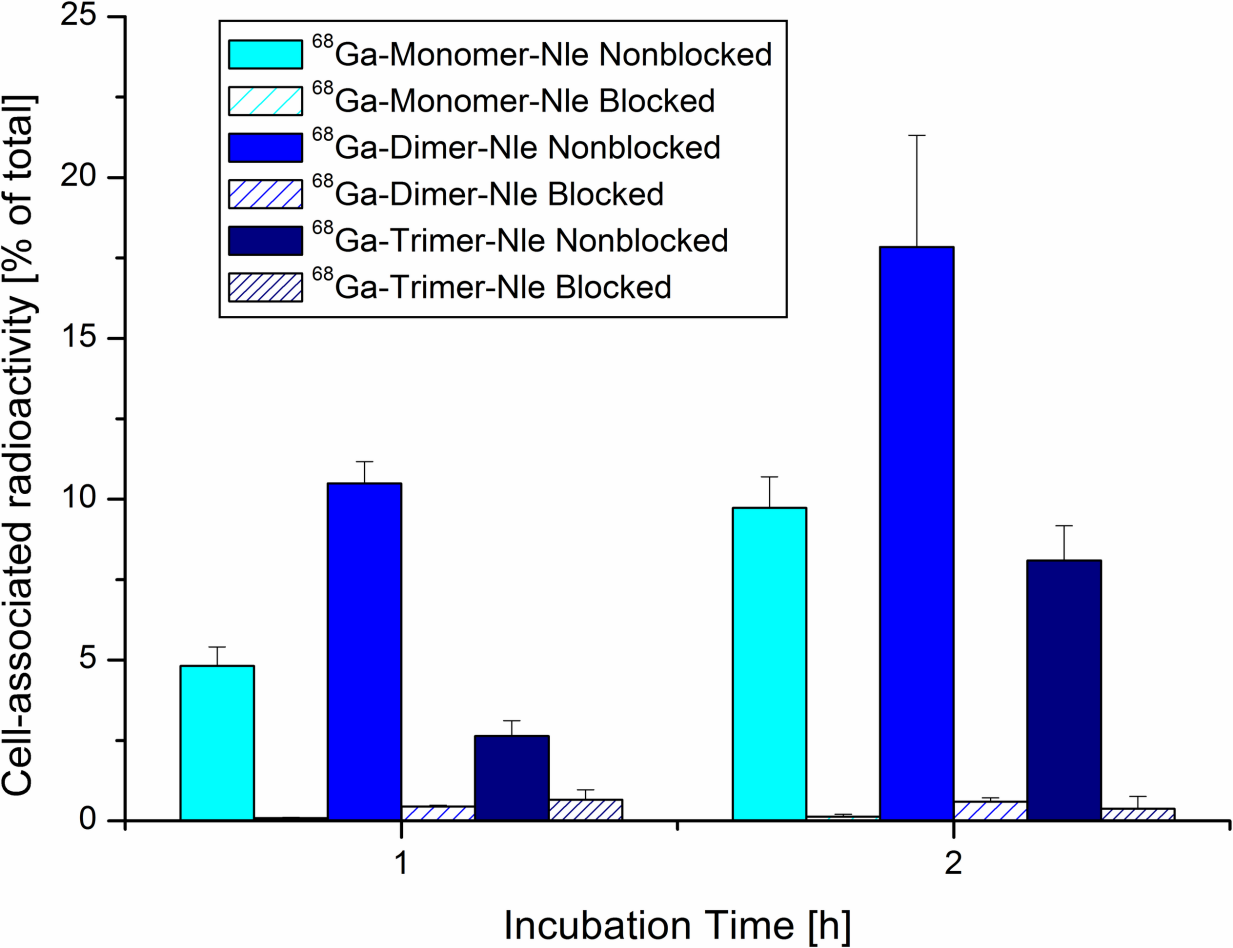
Cell-uptake studies of radiopeptides on CCK2R positive cells. Internalization studies of A431-CCK2R cells incubated with ^68^Ga-labelled mono- and multimeric Nle-derivatives. Blocking was performed with pentagastrin in 100-fold molar excess over the conjugate.

**Fig 4:**
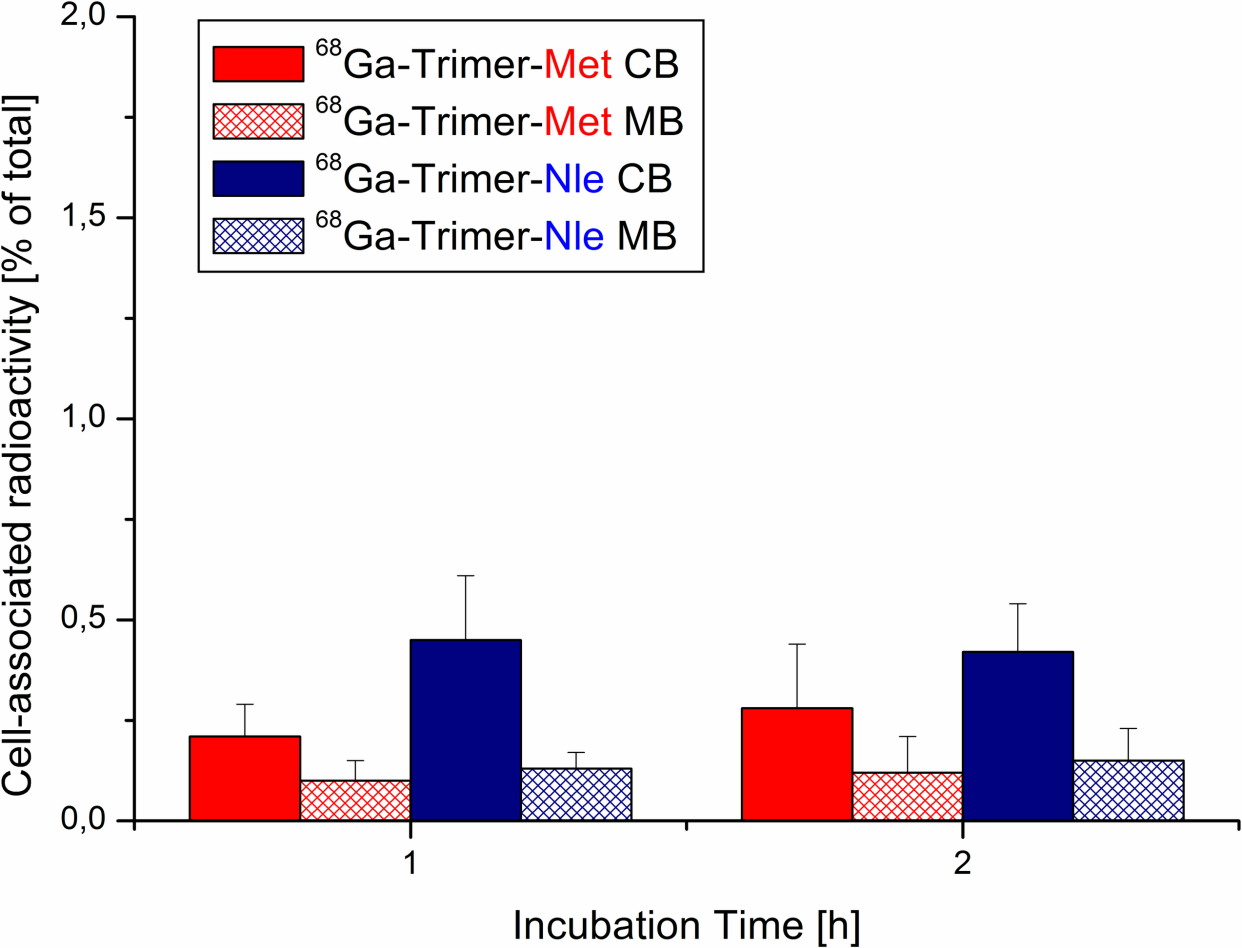
Cell-binding studies of ^68^Ga-labelled trimers on CCK2-mock negative cells. Cell-uptake studies of A431-mock cells incubated with ^68^Ga-trimer-Met and ^68^Ga-trimer-Nle (MB = membrane bound; CB = cell bound).

The results of internalization studies of ^68^Ga-labelled Nle-peptides on A431-CCK2R cells are presented in Fig 3 revealing highly specific receptor mediated uptake. As all conjugates showed increasing uptake over time while unspecific cell bound amount of radioligand remained < 1% of corresponding blocking studies. Furthermore, the ^68^Ga-dimer-Nle showed significantly increased uptake after 1h-incubation compared to the monomer (*P* = 6.67 × 10^−8^). This is in excellent agreement with earlier findings on ^68^Ga-labelled Met-peptides[15]. Except from ^68^Ga-dimer-Nle cell-uptake was generally significantly lower compared to the Met-counterparts and may be associated to the lower receptor affinity of corresponding Nle-conjugates. Exchange of Met by Nle resulting in modest decrease of affinity has been reported before and is in good agreement with our findings[19]. Receptor specificity was further substantiated with internalization studies of ^68^Ga-labelled trimers on A431-mock (receptor negative) cells as unspecific membrane as well as cell bound fraction was < 0.5% (Fig 4). These studies were only performed for trimeric targeting probes as they are supposed to have the highest unspecific cell binding due to their increased size and lipophilicity.

**Fig 5:**
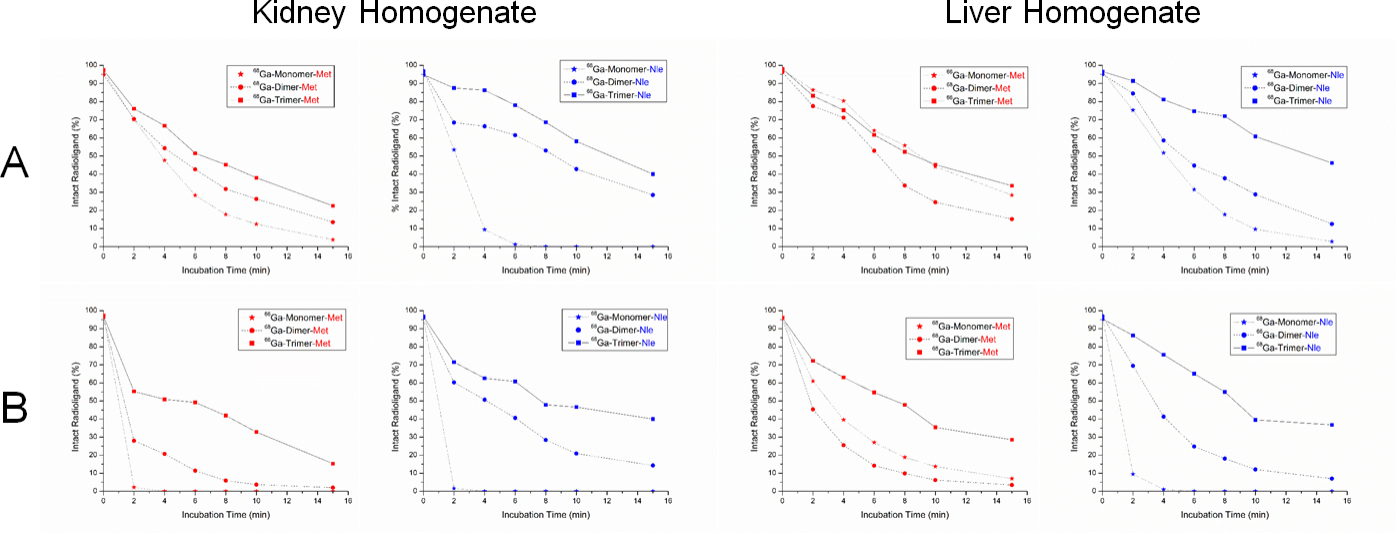
*In vitro* stability assessment of radiopeptides in rat organ homogenates. ^68^Ga-labelled mono- and multimeric peptide conjugates incubated with varying concentrations, 1% (A) and 10% (B) respectively, of rat kidney and liver homogenates. Data are presented as mean (n = 2), error bars omitted.

Stability studies in metabolic active tissue, rat liver and kidney homogenates respectively, are presented in Fig 5. The concentration of the homogenate affected the rate of degradation which is in good agreement with earlier investigations on the stability of DOTA-MG derivatives[20]. Furthermore, the trimeric conjugates showed to be less affected by homogenates especially at higher concentrations strongly indicating higher metabolic stability. However, as homogenisation disrupts the cells which additionally releases compartmentalized enzymes which are of less significance due to limited contact *in vivo*, these results do not fully represent the situation encountered *in vivo* where contact is restricted to membrane bound enzymes, particularly for liver and kidney tissue, and therefore should be interpreted with caution.

#### Investigations on metabolites formed *in vitro*

**Fig 6:**
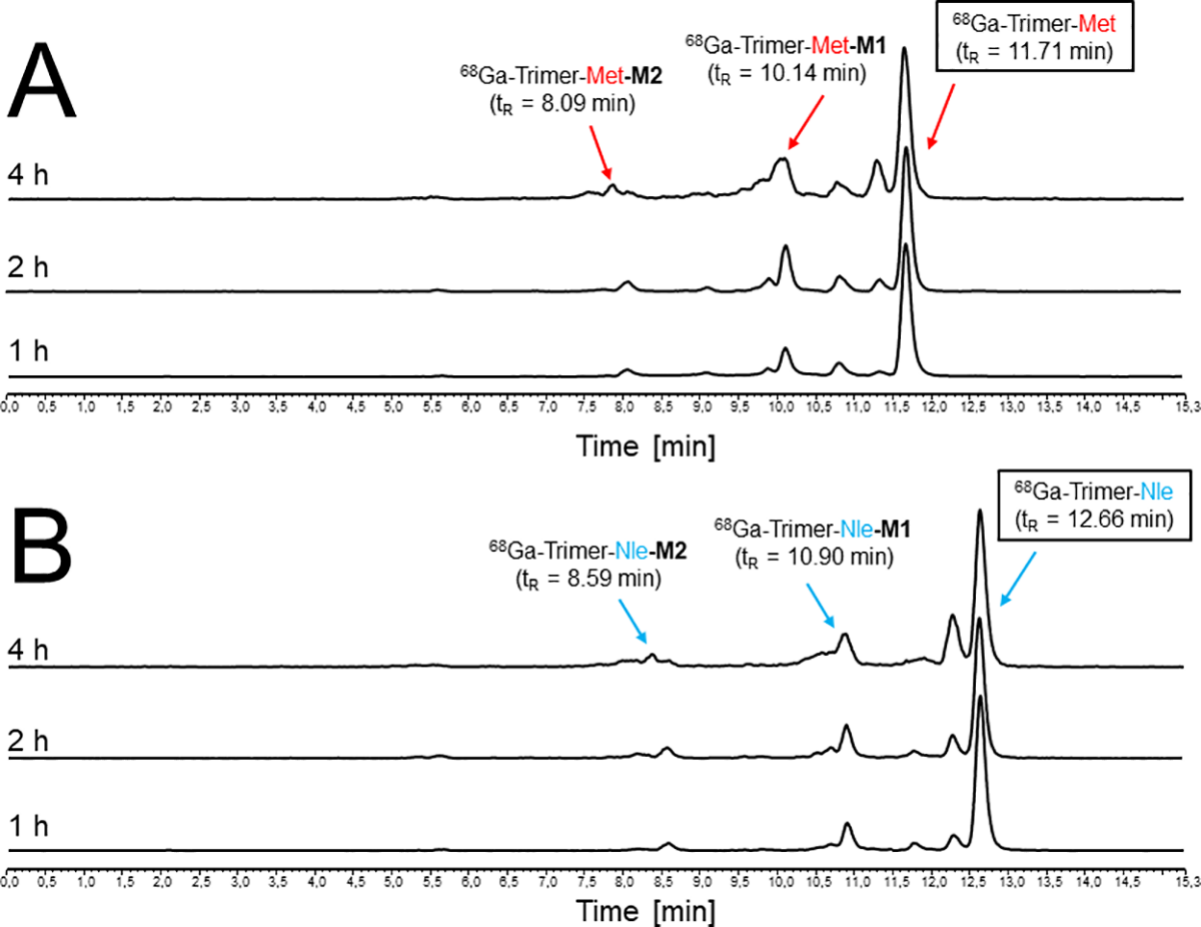
*In vitro* stability assessment of ^68^Ga-labelled trimers in fresh human serum. Radio-RP-HPLC analysis of the supernatant of ^68^Ga-labelled trimeric Met- (A) and Nle (B) conjugates incubated in fresh human serum. Major degradation products representing *in vitro* metabolites are assigned with arrows.

Incubating the ^68^Ga-labelled trimers in fresh human serum showed a slow formation of two major degradation products and the results of this study are presented in Fig 6. For artificial generation of the corresponding metabolites *in vitro* repeating studies were performed with radiolabelled as well as non-radioactive metal chelate variants of both trimers by incubation with peptidase. Although the peptidase used for this purpose is non-gastrin specific it allowed to generate metabolites in a standardized manner as it has been described in a previous study on DOTA-MG derivatives where the same enzyme was used to assess enzymatic stability[20].

**Fig 7:**
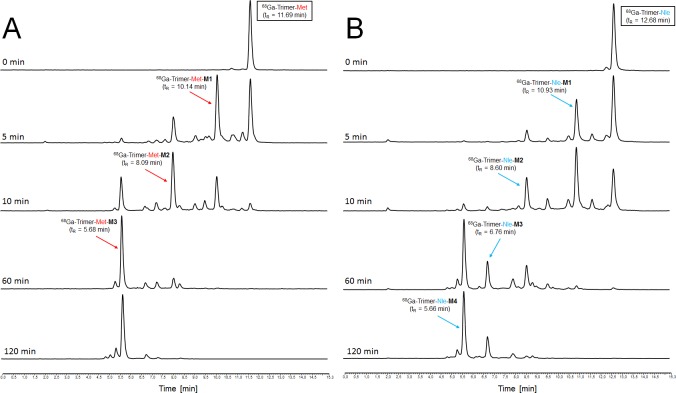
*In vitro* metabolite assessment of ^68^Ga-labelled trimers by artificial enzymatic degradation. Representative radio-RP-HPLC chromatograms of enzymatic degradation of ^68^Ga-labelled trimeric Met- (A) and Nle (B) conjugates *in vitro* (major metabolite formation assigned with arrows).

Investigations on the artificial enzymatic degradation profile of trimeric conjugates radiolabelled with gallium-68 are summarized in Fig 7. Both radiopeptides showed a very similar degradation profile with two major metabolites (M1 and M2 in order to their appearance to the starting material) formed over time to yield in a final cleavage product (M3, t_R_ = 5.66) after 2h-incubation which is neither ^68^Ga-FSC (t_R_ = 4.34) nor ^68^Ga-FSC-mal_3_ (t_R_ = 6.53) because of mismatch regarding retention time (radio-RP-HPLC shown in S1 Fig). It also should be mentioned that the retention time of M1 and M2 of both conjugates was similar to those of the corresponding ^68^Ga-labelled mono- and divalent targeting probe (Table 3) indicating that M1 might still have two and M2 might still provide one intact targeting moiety.

**Table 3:** Summary of retention time (t_R_) of radioactive and non-radioactive metabolites as well as ^68^Ga-labelled corresponding mono- and divalent imaging probes.

	^68^Ga-Monomer-Met	^68^Ga-Dimer-Met	^68^Ga-Trimer-Met-M1	^68^Ga-Trimer-Met-M2	Fe-Trimer-Met-M1	Fe-Trimer-Met-M2	Ga-Trimer-Met-M1	Ga-Trimer-Met-M2
t_R_ (min)	8.35	10.50	10.14	8.09	10.05	8.00	10.08	8.02
	^68^Ga-Monomer-Nle	^68^Ga-Dimer-Nle	^68^Ga-Trimer-Nle-**M1**	^68^Ga-Trimer-Nle-**M2**	Fe-Trimer-Nle-**M1**	Fe-Trimer-Nle-**M2**	Ga-Trimer-Nle-**M1**	Ga-Trimer-Nle-**M2**
t_R_ (min)	8.94	11.28	10.93	8.60	10.87	8.53	10.86	8.52

**Fig 8:**
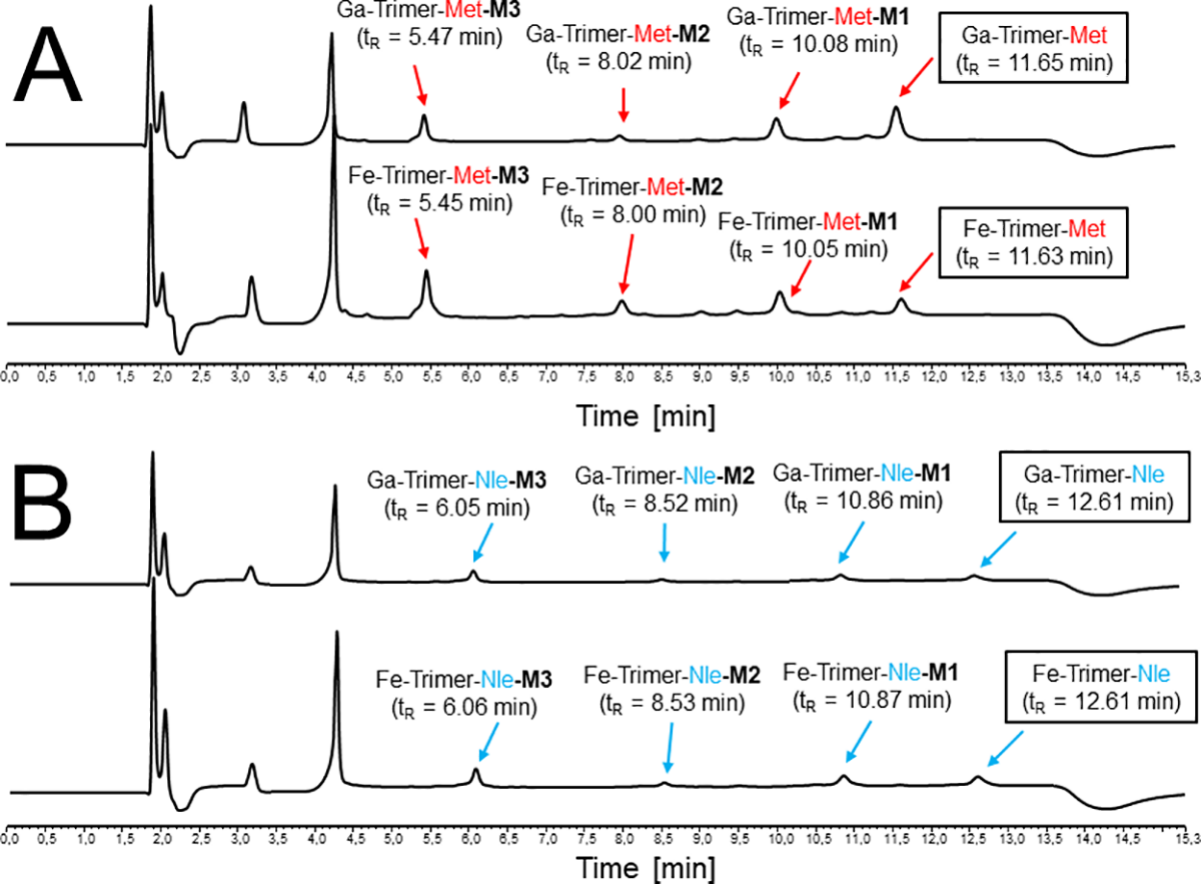
*In vitro* metabolite assessment of non-radioactive trimers by artificial enzymatic degradation. Representative RP-HPLC chromatograms of artificial enzymatic degradation *in vitro* of metal chelated trimeric Met- (A) and Nle (B) conjugates.

**Fig 9:**
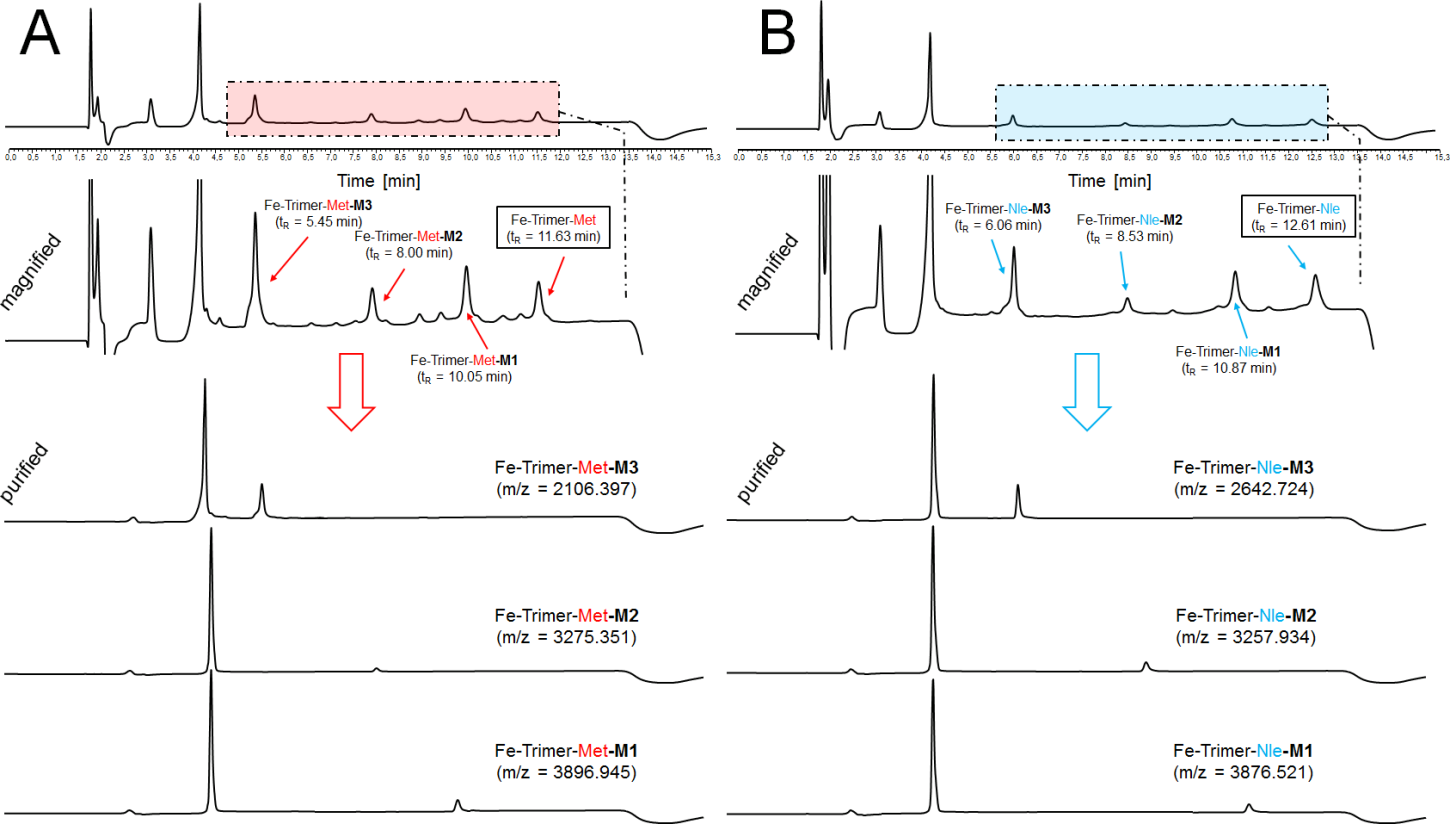
*In vitro* metabolite assessment, purification and identification of non-radioactive metabolites formed by artificial enzymatic degradation. Representative RP-HPLC chromatograms (UV absorption at λ = 220 nm) of enzymatic degradation of Fe-Trimer-Met (A) and Fe-Trimer-Nle (B). Below the arrow chromatograms of purified main metabolites are shown including molecular weight of corresponding mass analysis.

**Fig 10:**
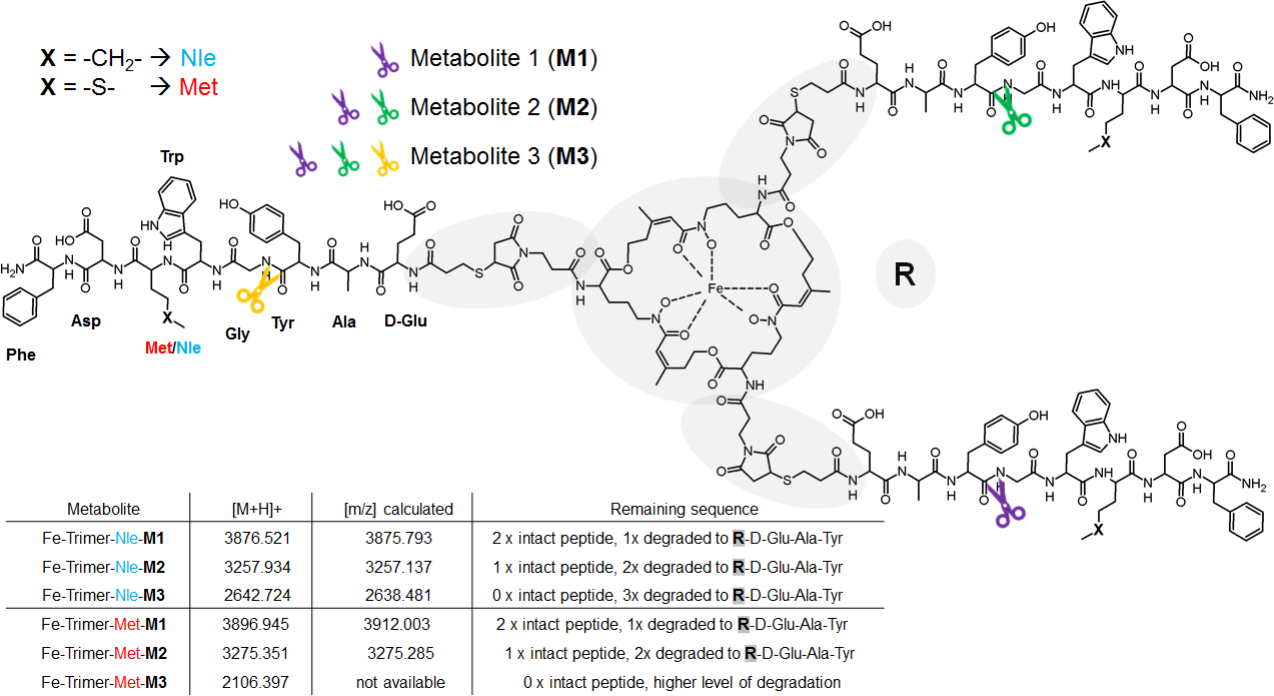
Propsed composition of metabolites calculated by computational assistance. Comparison of molecular weight of metabolites (measured vs. calculated) and proposed structure of metabolites including major cleavage site.

Repeating experiments with non-radioactive but gallium or iron chelated trimeric conjugates showed the same degradation profile with formed metabolites exhibiting corresponding retention times (Fig 8).Retention time of metabolites from Ga- and Fe-Trimer degradation are also given in Table 3 and minor changes in retention time as compared to ^68^Ga-counterparts is related to void volume effect due to the serial setup of UV detector followed by the radio-detector. For metabolite identification the degradation products of the Fe-trimers were used and the chromatograms of the isolated metabolites and the results of corresponding mass analysis are provided in Fig 9. The identified mass of the metabolites was taken for computer assisted calculation of all possible combinations of the remaining peptide sequence (calculation output is presented in detail in S1 Table) and a higher deviation of mass (±10 Da) was allowed to ensure to cover all variants within this error range. The results summarized in Fig 10 provide strong evidence that the formed metabolites, M1 and M2 respectively, still have intact MG peptides with the potential to bind to the CCK2 receptor while M3 does not. Even though there are more possible variants fitting the mass found for M3, they all showed no intact sequence and therefore M3 may represents the major degradation product which may be excreted via urinary pathway *in vivo*. Furthermore, the major metabolic cleavage site identified in this study between, Tyr^n^ and Gly^n^, is substantially supported and in excellent agreement with prior results on metabolic degradation of monovalent DOTA-MG derivatives[21].

### *In vivo* characterization

**Fig 11:**
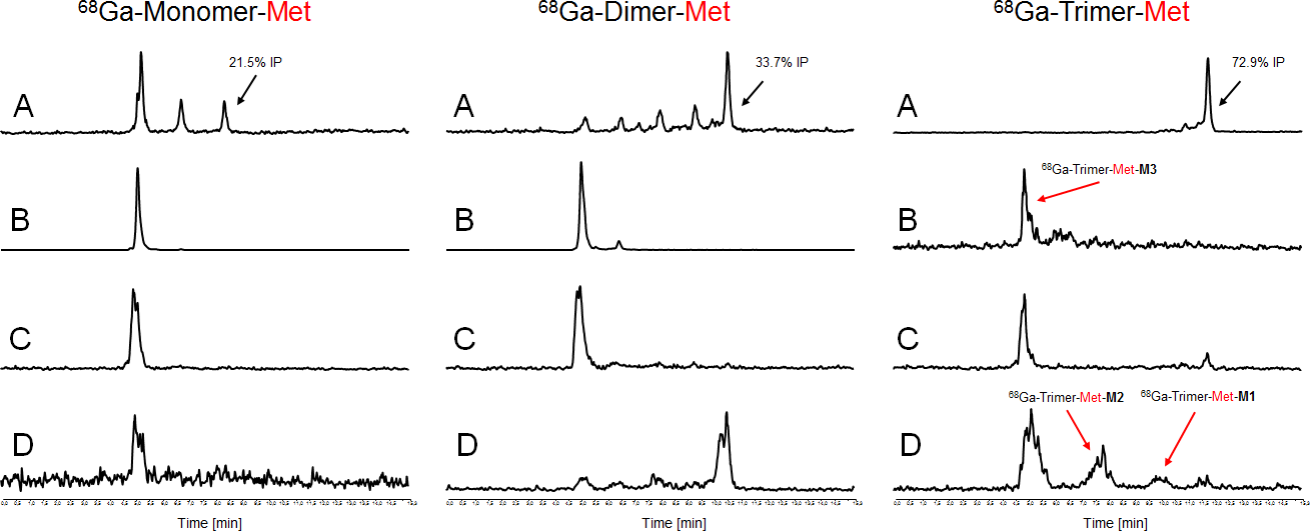
*In vivo* stability of ^68^Ga-labelled mono- and multimeric Met-MG based conjugates. Representative radio-RP-HPLC chromatograms of supernatants analysed from *in vivo* samples, serum (A), urine (B), kidney- (C) and liver homogenate (D). (IP = intact peptide).

**Fig 12:**
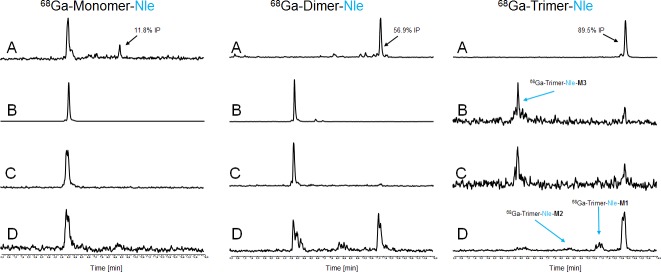
*In vivo* stability of ^68^Ga-labelled mono- and multimeric Nle-MG based conjugates. Representative radio-RP-HPLC chromatograms of supernatants analysed from *in vivo* samples, serum (A), urine (B), kidney- (C) and liver homogenate (D). (IP = intact peptide).

The results of *ex vivo* biodistribution studies in nude mice xenografted with receptor positive- and negative tumours are provided in Table 4. Corresponding calculated tumour-to-organ ratios are shown in Table 5. In general ^68^Ga-labelled Nle-conjugates showed lower uptake in malignant tissue, slower blood clearance (except from Monomer-Nle), lower renal accumulation and higher uptake in liver tissue compared to the corresponding Met-conjugates which can be attributed to the higher ability to bind to serum proteins, the decreased receptor affinity and the increased lipophilic character of the Nle-derivatives. Among all Nle-conjugates the divalent probe showed the highest accumulation in tumour tissue 1 h p.i. but corresponding tumour-to-organ ratios do not support superiority of multimeric probes for imaging at early time points, 1 h p.i. respectively. Still the trimeric probe showed increasing uptake after 4 h p.i. This correlates to our prior findings on mono- and multimeric Met-derivatives[15] where additional imaging studies with ^89^Zr-labelled mono- and multimeric Met peptides clearly demonstrated that after 24 h p.i. the trimer showed much lower tumour washout and reasonable imaging abilities while the corresponding mono- and dimer did not and it is highly likely to find a similar behaviour for the radiolabelled trimer-Nle although imaging was not performed in this study. *In vivo* stability studies presented in Figs 11 and 12 showed a degradation profile in order monomer > dimer > trimer in blood samples of living animals. Furthermore, liver homogenates indicated the appearance of the two main metabolites (M1 and M2) while M3 was mainly found in urine and kidney tissue having the same retention time as those metabolites identified by *in vitro* studies. The improved metabolic stability of the multimers taken together with the increased tumour uptake over time and improved late time point imaging with ^89^Zr-labelled counterparts strongly supports our hypothesis that degradation of multimers *in vivo* forms metabolites still being capable of binding to the CCK2 receptor.

**Table 4:** *Ex vivo* biodistribution studies of ^68^Ga-labelled mono- and multimers in A431-CCK2R/A431-mock tumour xenografted BALB/C nude mice; data are presented as percentage of injected activity per gram tissue (% IA/g); mean (n = 4) ± SD.

	Monomer-Met[15]	Dimer-Met[15]	Trimer-Met[15]		Monomer-Nle	Dimer-Nle	Trimer-Nle	
	1 h	1 h	1 h	4 h	1 h	1 h	1 h	4 h
Blood	0.35 ± 0.06^*ab*^	0.53 ± 0.06^*cd*^	3.70 ± 1.27^*d*^	1.76 ± 0.43^*de*^	0.25 ± 0.07^*ab*^	1.40 ± 0.48^*c*^	6.50 ± 1.23	4.45 ± 1.25
Spleen	0.18 ± 0.04^*ab*^	0.37 ± 0.04^*cd*^	2.15 ± 0.32^*d*^	2.77 ± 0.78^*d*^	0.10 ± 0.03^*ab*^	0.95 ± 0.07^*c*^	18.8 ± 2.85	12.9 ± 2.46^*e*^
Pancreas	0.29 ± 0.02^*ab*^	0.48 ± 0.06^*cd*^	1.37 ± 0.26	3.10 ± 1.13	0.24 ± 0.02^*abd*^	0.98 ± 0.28	1.88 ± 0.72	2.91 ± 1.15
Stomach	1.07 ± 0.14^*ab*^	2.13 ± 0.23^*c*^	3.17 ± 0.54	13.6 ± 4.9	0.66 ± 0.11^*abd*^	2.25 ± 0.17	4.12 ± 1.25	5.58 ± 1.64
Intestine	0.58 ± 0.17^*b*^	0.44 ± 0.06^*c*^	1.15 ± 0.28	2.48 ± 0.59	0.36 ± 0.12^*ab*^	0.64 ± 0.12^*c*^	1.33 ± 0.28	1.80 ± 0.35
Kidneys	5.57 ± 0.57^*ab*^	28.9 ± 3.4^*c*^	49.7 ± 10.1^*e*^	172.1 ± 14.8	2.72 ± 0.29^*abd*^	12.8 ± 0.16^*d*^	24.6 ± 8.59^*de*^	60.3 ± 10.7^*d*^
Liver	0.23 ± 0.02^*ab*^	0.78 ± 0.09^*cd*^	3.71 ± 0.75^*de*^	6.01 ± 2.54^*d*^	0.17 ± 0.01^*abd*^	3.49 ± 0.94^*c*^	53.6 ± 4.52	43.0 ± 11.3
Heart	0.17 ± 0.02^*ab*^	0.31 ± 0.04^*cd*^	1.60 ± 0.28^*d*^	1.51 ± 0.33	0.12 ± 0.03^*ab*^	0.62 ± 0.12^*c*^	2.89 ± 1.01	2.46 ± 1.06
Lung	0.42 ± 0.06^*ab*^	0.59 ± 0.08^*cd*^	4.47 ± 1.50^*d*^	3.75 ± 0.84^*e*^	0.25 ± 0.03^*abd*^	1.54 ± 0.33^*c*^	8.48 ± 1.31	4.40 ± 1.05^*e*^
Muscle	0.20 ± 0.05^*b*^	0.30 ± 0.08^*c*^	0.75 ± 0.15^*d*^	1.01 ± 0.37	0.10 ± 0.02^*ab*^	0.36 ± 0.12^*c*^	1.21 ± 0.32	0.96 ± 0.21
Bone	0.31 ± 0.08^*b*^	0.60 ± 0.21^*c*^	2.03 ± 0.88^*d*^	2.26 ± 0.41	0.30 ± 0.12^*b*^	0.80 ± 0.37^*c*^	3.89 ± 0.40	3.68 ± 1.52
A431-CCK2R	4.86 ± 1.00^*d*^	8.36 ± 0.90^*acd*^	6.07 ± 1.56	14.44 ± 3.40^*de*^	1.97 ± 0.25^*ab*^	5.00 ± 0.79	4.75 ± 0.76	7.63 ± 1.76^*e*^
A431-mock	0.30 ± 0.04^*b*^	0.36 ± 0.22^*c*^	2.17 ± 0.70	2.16 ± 0.54	0.22 ± 0.07^*ab*^	0.65 ± 0.14^*c*^	2.55 ± 0.56	3.11 ± 0.24

Statistical analysis was performed using the Student’s t-test with P values indicating significant (P < 0.05) difference (*a*) between mono- and dimer, (*b*) between mono- and trimer, (*c*) between di- and trimer, (*d*) between Met and Nle containing counterparts, (*e*) between 1h and 4h of radiolabelled trimer.

**Table 5:** Corresponding tumour-to-organ ratios of methionine (Met) and norleucine (Nle) containing FSC-based MG mono- and multimers radiolabelled with gallium-68, data are presented as mean ± SD.

	Monomer-Met[15]	Dimer-Met[15]	Trimer-Met[15]		Monomer-Nle	Dimer-Nle	Trimer-Nle	
Ratio T/O	1 h	1 h	1 h	4 h	1 h	1 h	1 h	4 h
Blood	14.4 ± 3.6^*b*^	16.0 ± 3.1^*cd*^	1.8 ± 0.5^*d*^	8.5 ± 2.1^*de*^	9.0 ± 3.6^*b*^	4.0 ± 1.3^*c*^	0.74 ± 0.07	1.8 ± 0.3^*e*^
Spleen	27.3 ± 6.3^*b*^	23.1 ± 4.2^*cd*^	2.8 ± 0.4^*d*^	5.5 ± 1.4^*de*^	19.8 ± 4.0^*ab*^	5.2 ± 0.5^*c*^	0.25 ± 0.02	0.6 ± 0.3^*e*^
Pancreas	16.7 ± 3.0^*bd*^	17.9 ± 3.2^*cd*^	4.5 ± 1.0^*d*^	4.7 ± 1.5	8.4 ± 1.6^*b*^	5.4 ± 1.3^*c*^	2.8 ± 0.7	2.8 ± 0.4
Stomach	4.6 ± 1.1^*b*^	4.0 ± 0.5^*cd*^	2.0 ± 0.8^*e*^	1.1 ± 0.3	3.1 ± 0.9^*b*^	2.2 ± 0.3^*c*^	1.2 ± 0.3	1.4 ± 0.3
Intestine	9.4 ± 3.7^*b*^	19.9 ± 4.9^*acd*^	5.4 ± 1.1^*d*^	6.0 ± 1.3	6.6 ± 3.6	8.1 ± 1.8^*c*^	3.6 ± 0.5	4.3 ± 0.7
Kidneys	0.86 ± 0.13^*ab*^	0.30 ± 0.06^*c*^	0.13 ± 0.05	0.07 ± 0.01	0.75 ± 0.18^*b*^	0.39 ± 0.06^*c*^	0.21 ± 0.06^*d*^	0.13 ± 0.01^*d*^
Liver	21.1 ± 3.1^*abd*^	11.1 ± 2.4^*cd*^	1.7 ± 0.6^*d*^	2.8 ± 1.0^*de*^	11.5 ± 2.0^*ab*^	1.5 ± 0.4^*c*^	0.09 ± 0.01	0.21 ± 0.11
Heart	29.5 ± 9.3^*b*^	27.2 ± 4.8^*cd*^	3.8 ± 1.0^*d*^	10.1 ± 3.2^*de*^	17.6 ± 5.2^*b*^	8.2 ± 1.2^*c*^	1.8 ± 0.4	3.6 ± 1.4
Lung	11.7 ± 2.1^*b*^	14.4 ± 2.3^*cd*^	1.5 ± 0.4^*d*^	3.9 ± 0.8^*de*^	8.1 ± 2.1^*ab*^	3.3 ± 0.5^*c*^	0.56 ± 0.07	1.7 ± 0.3^*e*^
Muscle	20.4 ± 1.8^*b*^	31.4 ± 13.2^*c*^	8.4 ± 2.6^*d*^	13.5 ± 5.4	22.1 ± 6.0^*b*^	15.8 ± 5.8^*c*^	4.1 ± 0.8	7.9 ± 0.3^*e*^
Bone	17.1 ± 7.4^*b*^	11.6 ± 1.9^*c*^	2.6 ± 1.0^*d*^	7.2 ± 2.6^*e*^	7.9 ± 3.1^*b*^	7.6 ± 3.2^*c*^	1.2 ± 0.2	2.8 ± 1.8
A431-mock	16.3 ± 3.3^*b*^	17.9 ± 6.1^*c*^	2.9 ± 0.4^*d*^	7.0 ± 1.9^*de*^	9.8 ± 2.2^*b*^	8.0 ± 1.8^*c*^	2.0 ± 0.5	2.5 ± 0.5

Statistical analysis was performed using the Student’s t-test with P values indicating significant (P < 0.05) difference (*a*) between mono- and dimer, (*b*) between mono- and trimer, (*c*) between di- and trimer, (*d*) between Met and Nle containing counterparts, (*e*) between 1h and 4h of radiolabelled trimer.

However, the high blood levels, in particular for the ^68^Ga-labelled trimers 1 h after administration indicate slow pharmacokinetics and thus are less favourable in terms of using the short-lived gallium-68 for imaging purposes. In comparison to a variety of monovalent DOTA-MG derivatives being investigated on reducing kidney but retain tumour uptake [22] this mutlivalency approach did not provide any advantages as high accumulation in renal tissue of the FSC-based multivalent conjugates is certainly unfavourable towards clinical translation for imaging of CCK2R related malignancies. Although the use of mutlivalency in this particular case seems to be limited, the results on prolonged stability and metabolites with retained interaction potential may contribute to other peptide based targets and areas of research.

## Conclusion

In this study we confirmed the feasibility of FSC as chelating scaffold for the synthesis of multimeric conjugates. Multimerization increased metabolic stability *in vitro* and *in vivo* of a metabolically unstable model peptide. Additionally the isolation and identification of the main degradation products confirm the hypothesis that multimerization also can result in formation of metabolites with retained binding affinity to the receptor. This effect can prolong targeting ability and improve image contrast. Multimerization therefore can, besides known effects on improved affinity and avidity, also prolong *in vivo* activity of otherwise too metabolically unstable bioconjugates for molecular imaging and therapy applications.

## Supporting information

S1 Fig Radiolabelling with gallium-68 of FSC and FSC derivatives without MG moieties. (TIF)

S1 Table Computer assisted calculation of metabolite composition. (DOCX)

## Abbreviations

FSC: fusarinine C
MG: minigastrin
Met: [3-MP^0^-D-Glu^1^,desGlu^2-6^]-Ala-Tyr-Gly-Trp-Met-Asp-Phe-NH_2_] MG sequence
Nle: [3-MP^0^-D-Glu^1^,desGlu^2-6^]-Ala-Tyr-Gly-Trp-Nle-Asp-Phe-NH_2_] MG sequence
CCK2: cholecystokinin receptor subtype 2
PET/CT: positron emission computed tomography, RT, room temperature
DOTA: 1,4,7,10-tetraazacyclododecane-1,4,7,10-tetraacetic acid
PBS: phosphate buffered saline
BSA: bovine serum albumin
HEPES: 4-(2-hydroxyethyl)-1-piperazineethanesulfonic acid buffer
TRIS: tris-(hydroxymethyl)aminomethane
EDTA: ethylenediaminetetra acetic acid
RP-HPLC: reversed phase high performance liquid chromatography
TLC: thin layer chromatography
p.i.: post injection
