# Supplementary figures and images for: Multimerization results in formation of re-bindable metabolites: A proof of concept study with FSC-based minigastrin imaging probes targeting CCK2R expression
Source: PLoS One. 2018 Jul 30;13(7):e0201224. doi: 10.1371/journal.pone.0201224 (PMC6066219; doi:10.1371/journal.pone.0201224)

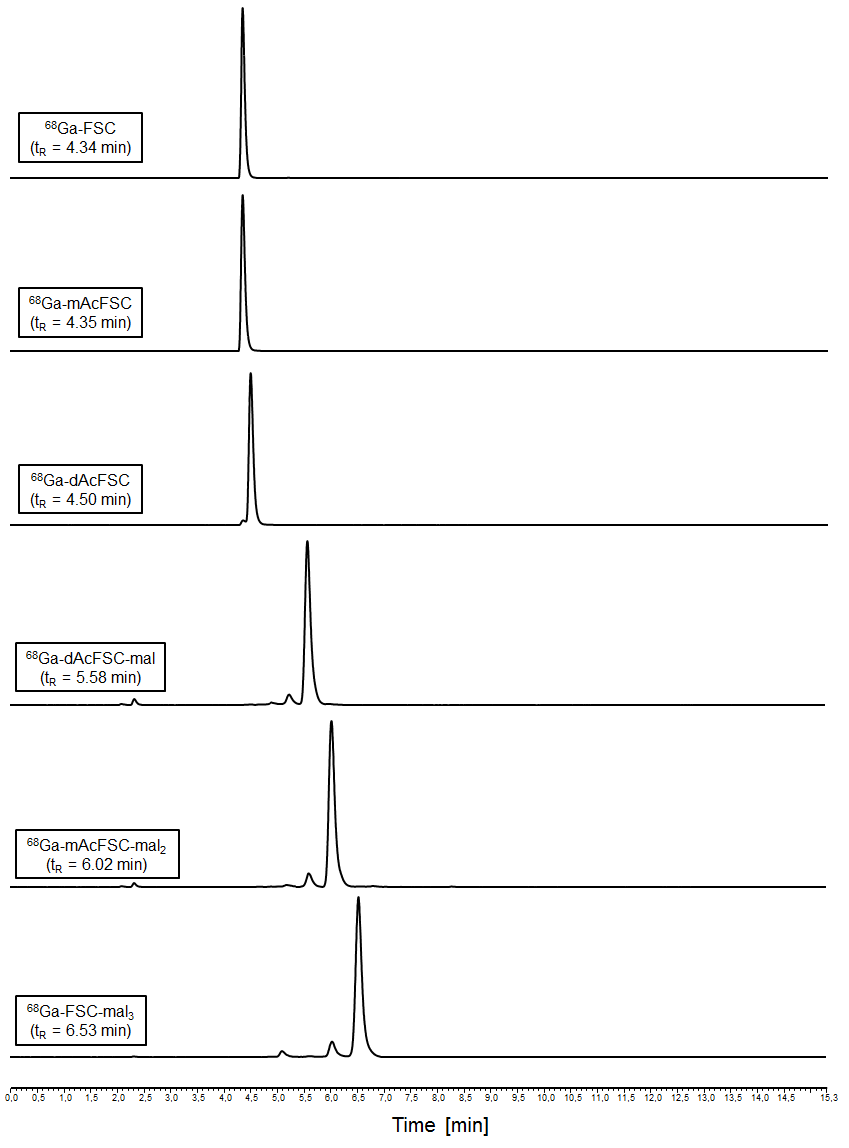

Supplement: S1 Fig — (TIF) [file pone.0201224.s001.tif]
