# Supplementary material for: Multimerization results in formation of re-bindable metabolites: A proof of concept study with FSC-based minigastrin imaging probes targeting CCK2R expression
Source: PLoS One. 2018 Jul 30;13(7):e0201224. doi: 10.1371/journal.pone.0201224 (PMC6066219; doi:10.1371/journal.pone.0201224)
Supplement: S1 Table — (DOCX) [file pone.0201224.s002.docx]

Supporting information for

Multimerization results in formation of re-bindable metabolites: a proof of concept study with FSC-based minigastrin imaging probes targeting CCK2R expression

Dominik Summer^1^, Andrea Kroess^1^, Rudolf Woerndle^1^, Christine Rangger^1^, Maximilian Klingler^1^, Hubertus Haas^2^, Leopold Kremser^3^, Herbert H. Lindner^3^, Elisabeth von Guggenberg^1^ and Clemens Decristoforo^1,*^

^1^Department of Nuclear Medicine, Medical University of Innsbruck, Innsbruck, Austria

^2^Division of Molecular Biology, Biocenter, Medical University of Innsbruck, Innsbruck, Austria

^3^Division of Clinical Biochemistry, Biocenter, Medical University of Innsbruck, Innsbruck, Austria

***Corresponding Author**

E-mail: Clemens.Decristoforo@i-med.ac.at

**S1 Fig.** **Radiolabelling with gallium-68 of FSC and FSC derivatives without MG moieties.** Representative radio-RP-HPLC chromatograms of ^68^Ga-labelled FSC-based chelating scaffolds without CCK2R targeting MG moieties.

**S1 Table.** **Computer assisted calculation of metabolite composition** (number below metals indicates appearance while number below amino acids assigns cleavage)

| Letter Code | Meaning | Letter Code | Meaning |
| --- | --- | --- | --- |
| Fe | iron | M | Methionine (Met) |
| Na | sodium | W | Tryptophan (Trp) |
| K | potassium | G | Glycine (Gly) |
| F | Phenylalanine (Phe) | Y | Tyrosine (Tyr) |
| D | Aspartic acid (Asp) | A | Alanine (Ala) |
| X | Norleucine (Nle) | E | D-Glutamic acid (D-Glu) |

List of Trimer-Nle metabolites

Name: Fe-Trimer-Nle-**M1**

Measure: 3876.5210

**+Fe -Fe +Na +K F D X W G Y A E**

[0, 1, 0, 0] [1, 1, 1, 1, 0, 0, 0, 0] Error: +3.5005 (3880.0215)

[0, 1, 0, 0] [3, 1, 0, 0, 0, 0, 0, 0] Error: +8.5208 (3885.0418)

**[1, 0, 0, 0] [1, 1, 1, 1, 1, 0, 0, 0] Error: -0.7280 (3875.7930)**

Solutions (within +/-10.0 tolerance): 3

Name: Fe-Trimer-Nle-**M2**

Measure: 3257.9340

**+Fe -Fe +Na +K F D X W G Y A E**

[0, 1, 0, 0] [2, 2, 2, 2, 1, 0, 0, 0] Error: +3.4314 (3261.3654)

[0, 1, 0, 0] [3, 2, 1, 1, 1, 1, 0, 0] Error: -7.5476 (3250.3864)

[0, 0, 0, 1] [2, 2, 2, 1, 1, 1, 1, 0] Error: -5.5109 (3252.4231)

[0, 0, 1, 0] [3, 3, 2, 1, 0, 0, 0, 0] Error: +7.4209 (3265.3549)

**[1, 0, 0, 0] [2, 2, 2, 2, 2, 0, 0, 0] Error: -0.7971 (3257.1369)**

[1, 0, 0, 0] [2, 2, 2, 1, 1, 1, 1, 0] Error: +8.2124 (3266.1464)

[1, 0, 0, 0] [2, 2, 1, 1, 1, 1, 1, 1] Error: -7.7447 (3250.1893)

Solutions (within +/-10.0 tolerance): 7

Name: Fe-Trimer-Nle-**M3**

Measure: 2642.7240

**+Fe -Fe +Na +K F D X W G Y A E**

[0, 1, 0, 0] [3, 3, 3, 2, 1, 1, 1, 0] Error: +8.9948 (2651.7188)

[0, 1, 0, 0] [3, 3, 2, 2, 1, 1, 1, 1] Error: -6.9623 (2635.7617)

[0, 1, 0, 0] [3, 3, 3, 3, 2, 0, 0, 0] Error: -0.0147 (2642.7093)

[0, 0, 0, 1] [2, 2, 2, 2, 2, 2, 2, 1] Error: +3.0955 (2645.8195)

[0, 0, 0, 1] [3, 3, 3, 2, 2, 1, 1, 0] Error: -8.9570 (2633.7670)

[0, 0, 1, 0] [3, 3, 2, 2, 2, 2, 0, 0] Error: -4.0034 (2638.7206)

**[1, 0, 0, 0] [3, 2, 2, 2, 2, 2, 2, 0] Error: -1.2383 (2641.4857)**

**[1, 0, 0, 0] [3, 3, 3, 2, 2, 1, 1, 0] Error: +4.7663 (2647.4903)**

**[1, 0, 0, 0] [3, 3, 3, 3, 3, 0, 0, 0] Error: -4.2432 (2638.4808)**

Solutions (within +/-10.0 tolerance): 9

List of Trimer-Met metabolites

Name: Fe-Trimer-Met-**M1**

Measure: 3896.9450

**+Fe -Fe +Na +K F D M W G Y A E**

**[0, 0, 0, 1] [1, 1, 1, 1, 1, 0, 0, 0] Error: +2.3623 (3899.3073)**

[1, 0, 0, 0] [2, 2, 1, 0, 0, 0, 0, 0] Error: -2.9145 (3894.0305)

Solutions (within +/-10.0 tolerance): 2

Name: Fe-Trimer-Met-**M2**

Measure: 3275.3510

**+Fe -Fe +Na +K F D M W G Y A E**

[0, 1, 0, 0] [2, 2, 2, 2, 1, 0, 0, 0] Error: +6.2168 (3281.5678)

[0, 0, 0, 1] [2, 2, 2, 1, 1, 1, 1, 0] Error: -2.7255 (3272.6255)

[0, 0, 0, 1] [2, 2, 1, 1, 1, 1, 1, 1] Error: -1.6474 (3273.7036)

**[1, 0, 0, 0] [2, 2, 2, 2, 2, 0, 0, 0] Error: +1.9883** (3277.3393)

[1, 0, 0, 0] [3, 2, 1, 1, 1, 1, 1, 0] Error: -5.9812 (3269.3698)

Solutions (within +/-10.0 tolerance): 5

Name: Fe-Trimer-Met-**M3**

Measure: 2106.3970

**+Fe -Fe +Na +K F D M W G Y A E**

**[0, 1, 0, 0] [3, 3, 3, 2, 2, 2, 2, 2] Error: -1.0230 (2105.3740)**

[0, 1, 0, 0] [3, 3, 3, 3, 3, 3, 0, 0] Error: -7.0698 (2099.3272)

[0, 0, 0, 1] [3, 3, 3, 3, 3, 2, 1, 1] Error: -4.9909 (2101.4061)

[1, 0, 0, 0] [3, 3, 3, 3, 2, 2, 2, 1] Error: -5.2933 (2101.1037)

[1, 0, 0, 0] [3, 3, 3, 3, 3, 2, 1, 1] Error: +8.7324 (2115.1294)

Solutions (within +/-10.0 tolerance): 5
